# Oral Intravascular Papillary Endothelial Hyperplasia Associated with an Organizing Thrombus: Case Report and Immunohistochemical Analysis

**DOI:** 10.1155/2016/1908767

**Published:** 2016-12-07

**Authors:** Darcy Fernandes, Daphine Caxias Travassos, Túlio Morandin Ferrisse, Elaine Maria Sgavioli Massucato, Cláudia Maria Navarro, Mirian Aparecida Onofre, Jorge Esquiche León, Andreia Bufalino

**Affiliations:** ^1^Department of Diagnosis and Surgery, Araraquara Dental School, Univ Estadual Paulista (UNESP), Araraquara, SP, Brazil; ^2^Oral Pathology, Department of Stomatology, Public Oral Health, and Forensic Dentistry, School of Dentistry of Ribeirão Preto (FORP), University of São Paulo (USP), Ribeirão Preto, SP, Brazil

## Abstract

Intravascular papillary endothelial hyperplasia (IPEH) is a benign lesion of the skin and mucosa of vascular origin characterized by reactive proliferation of endothelial cells. A 76-year-old woman was referred presenting a painless nodule on the lip. Intraoral examination revealed bluish submucosal nodular proliferation, measuring 10 × 5 × 5 mm, affecting the lower labial mucosa. The lesion had a firm consistency and it was not fixed to the adjacent tissues. The main differential diagnoses were mucocele/mucus retention cyst, sialolith, or salivary gland neoplasia. An incisional biopsy was performed and during the intraoperative procedure an encapsulated red-bluish nodular mass was observed. Microscopic analysis revealed papillary endothelial proliferation in the center of the lesion and fibrin admixed with inflammatory cells in organization peripherally. There was no nuclear atypia, mitotic figures, or necrosis. The endothelial cells were CD34 positive, with low Ki-67 proliferation index (4%). *α*-SMA highlighted the vessel walls, whereas negativity for D2-40 excluded lymphatic origin. Final diagnosis was IPEH associated with an organizing thrombus. Dentists should be aware about this rare benign vascular lesion, whose final diagnosis is achieved only after histopathology analysis. Surgical removal is the treatment of choice and no recurrence is expected.

## 1. Introduction

Intravascular papillary endothelial hyperplasia (IPEH) was first described by Masson in 1923 [1]. It is a benign lesion of the skin, mucosa, and subcutaneous tissue consisting of reactive proliferation of endothelial cells with papillary formations related to an abnormal process of organization in thrombosed blood vessels [2, 3]. IPEH's etiology is unknown and comprises approximately 2% of all the vascular proliferation cases of the skin and subcutaneous tissues [4]. The most common sites of IPEH are head and neck region, fingers, and trunk, but these lesions may occur in any blood vessel. When IPEH lesions are large and show some degree of cellular proliferation, it may be difficult to differentiate them from low-grade angiosarcoma. We report a case of IPEH affecting the oral cavity and discuss the clinical and histopathological findings. To our knowledge, to date, approximately 118 [5, 6] oral IPEH cases were reported in the English-language literature.

## 2. Case Report

**Figure 1:**
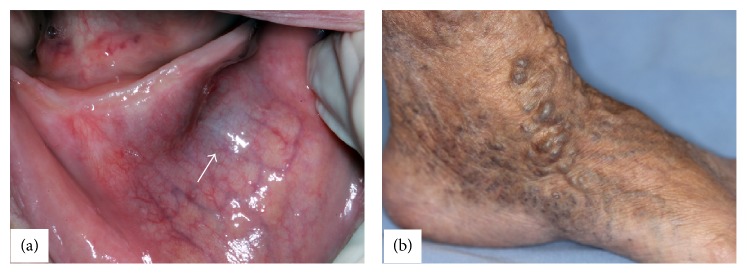
Clinical appearance of the submucosal nodule on the lower labial mucosa (arrow) (a). The same patient presented numerous varices of irregular shape and variable size in the foot (b).

**Figure 2:**
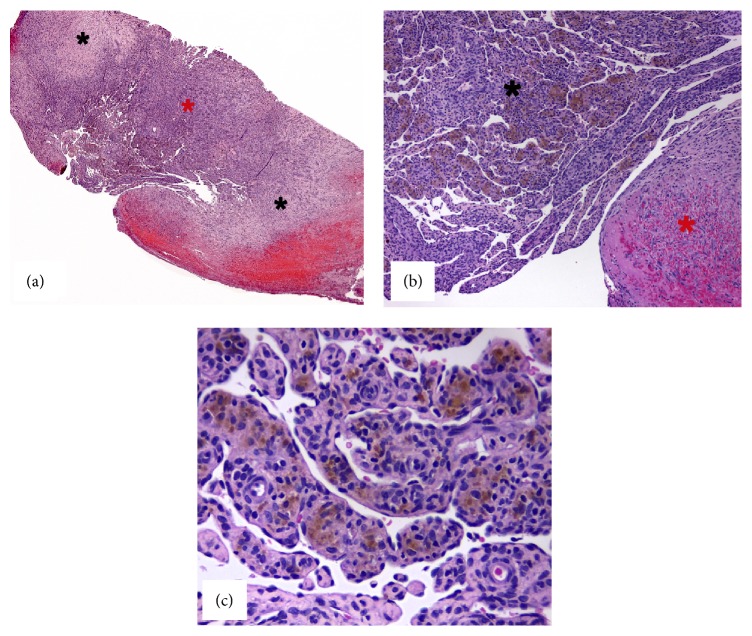
Histopathological findings of IPEH. (a) Photomicrograph showing thrombus in organization in the peripheral region (black stars) and intravascular papillary endothelial proliferation in the central region (red star) (H&E, ×4). (b) Close relationship between papillae structures in association with hemosiderin deposition (black star) and the thrombus (red star) (H&E, ×10). (c) Close-up of reactive papillary endothelial proliferation containing hemosiderin deposits (H&E, ×40).

**Figure 3:**
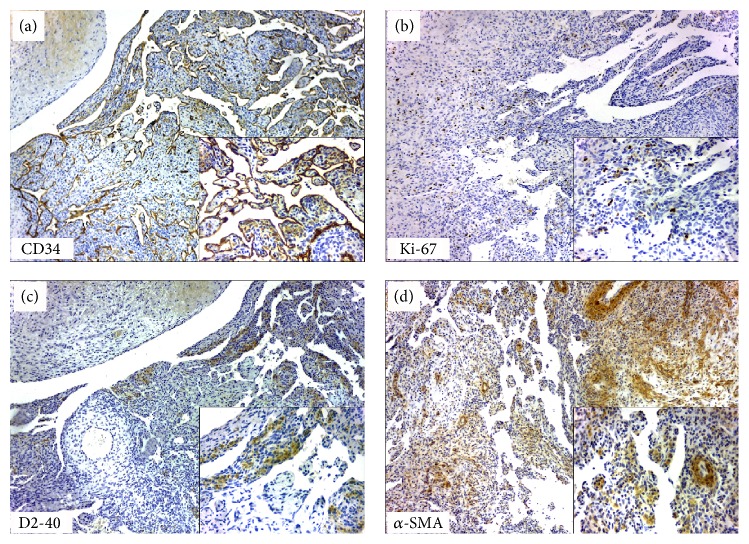
Immunohistochemical findings. CD34 was strongly positive highlighting the papillary endothelial proliferation (a) with low Ki-67 proliferation index (4%) (b). D2-40 was negative (notice the hemosiderin deposits) (c), whereas *α*-SMA stained the vessel walls (d) (all figures, ×10; all insets ×40).

A 76-year-old white woman was referred to the Oral Medicine Service for evaluation of a nodule of 2-week duration; medical history revealed rheumatoid arthritis and hypercholesterolemia. The only extraoral finding was the presence of varices on feet and legs. At intraoral examination, a bluish firm nodule on the lower labial mucosa, painless, measuring 10 × 5 × 5 mm, and covered by normal mucosa, was observed (Figure 1). The lesion was biopsied and during the surgery encapsulated dark red nodular proliferation was visualized. The histopathological examination revealed papillary endothelial proliferation and hemosiderin deposits on central region, as well as the presence of fibrin and inflammatory cells in organization on peripheral region. No cellular or nuclear atypia, abnormal mitosis, or necrosis was present (Figure 2). By immunohistochemical analysis, the endothelial cells revealed positivity for CD34 and low Ki-67 proliferation index (4%). D2-40 was negative, whereas alpha-smooth muscle antigen (*α*-SMA) highlighted the smooth muscle cells in vessel walls (Figure 3). Clinical presentation, histopathological findings, and immunohistochemistry confirmed the diagnosis of IPEH associated with thrombus in organization. Six months after surgical resection, the patient is well, with no complications or evidence of recurrence.

## 3. Discussion

Clinical presentation of oral IPEH is nonspecific and it may mimic a variety of lesions such as mucocele, mucous retention cyst, hemangioma, salivary gland tumor, sialolith, or benign mesenchymal neoplasm such as myofibroma, neurofibroma, schwannoma, or leiomyoma. Females are slightly more affected than males (ratio 1.14 : 1) and the oral site most frequently involved is the lower lip (40.5%), although nearly any other oral site can be affected [6]. IPEH usually manifests as a soft to firm painless mass, sometimes tender, ranging in size from 0.5 to 1.8 cm in diameter and imparts a reddish blue color to the overlying skin or mucous membrane [6–8]. An interesting finding in the current case was the presence of high-caliber varices in feet and legs that might demonstrate association with other vascular malformations. However, there are no reports that correlate these two disorders; thus, further investigation is recommended.

Various other benign and malignant vascular proliferative disorders should be considered in the histopathological differential diagnosis; in particular the papillae fuse to form an anastomotic vascular pattern that may mimic the appearance of angiosarcoma [9–12]. Nonetheless, IPEH is characterized by papillary proliferation of reactive endothelial cells. The papillae structures consist of hypocellular and hyalinized cores covered or lined by one or two layers of plump endothelium commonly associated with thrombus. Most importantly, IPEH lacks cellular atypia, atypical mitosis, and an infiltrative pattern of growth; all these important features are often indicative of malignancy. In fact, low-grade angiosarcoma presents infiltrative growth pattern, as well as nuclear hyperchromatism, mitosis, cellular atypia, and necrosis [3, 8]. Three different types of IPEH have been reported: (a) a primary (pure) form where changes are observed in a distended vessel; (b) a secondary (mixed) form that occurs in preexisting varices, hemangiomas, pyogenic granulomas, or lymphangiomas; and (c) an uncommon type in an extravascular location [11]. In the current case, a large lesional area was observed; the peripheral region of the lesion was not well-defined and in close contact with a discrete band of fibrocellular connective tissue, suggesting a preexisting varix, and consequently supporting the secondary form.

Some immunohistochemical markers can be useful in the identification of the IPEH lesions. Moreover, the Ki-67, a cellular marker for proliferation, may help to predict the biological behavior of IPEH lesions [6]. Usually the Ki-67 index labelling is low (<10%), and, apparently, cases that show higher immunopositivity tend to recur more often [3, 13]. Moreover, for better characterizing of IPEH, several other endothelial cell markers were already used as CD34 (pan-endothelial marker), CD31 (platelet endothelial cell adhesion molecule-1), and CD105 (endoglin) [3, 12, 13]. Differently from CD34 and CD31 immunomarkers, the CD105 immunostaining is useful in the differential diagnosis since it is overexpressed in angiosarcoma cells [3]. Vimentin, laminin, podoplanin, and type IV and type I collagen immunopositivity were also reported, highlighting the complexity of the stroma in these lesions [12, 13]. The current case showed CD34 positivity in the endothelial cells and low Ki-67 proliferation index (4%). Moreover, *α*-SMA highlighted the vessel walls, whereas D2-40 (podoplanin) was negative. This latter excluded a lymphatic origin in the current IPEH case.

The treatment of choice for IPEH is total excision, and recurrence is extremely rare [7]. Sclerotherapy prior to surgery might be useful in mixed type cases considering that significant bleeding can happen during intraoperative period [10].

## 4. Conclusion

As mentioned above, the clinical features of IPEH are nonspecific; hence, the histopathological examination, sometimes supplemented by immunohistochemistry, is required for establishing a correct diagnosis. Regarding its histopathological resemblance to low-grade angiosarcoma, oral IPEH lesions should be recognized, especially by dentists, preventing misdiagnosis and unnecessary aggressive treatment.
